# Assessment of General and Sports Nutrition Knowledge, Dietary Habits, and Nutrient Intake of Physical Activity Practitioners and Athletes in Riyadh, Saudi Arabia

**DOI:** 10.3390/nu15204353

**Published:** 2023-10-12

**Authors:** Alaa K. Alahmadi, Reem S. Albassam

**Affiliations:** Department of Community Health Sciences, College of Applied Medical Sciences, King Saud University, Riyadh 11451, Saudi Arabia; alaakhalidalahmadi@gmail.com

**Keywords:** physical activity practitioners, athletes, nutritional knowledge, sports nutrition, dietary intake

## Abstract

Physical performance and overall health are crucial in the athletic population, and their improvement relies on specific dietary guidelines and practices. Athletes and physical activity practitioners who participate in physical activity for specific health reasons need nutritional knowledge to improve physical performance. This study aimed to understand nutrient intake and nutritional knowledge among physical activity practitioners and athletes in Riyadh, Saudi Arabia. This cross-sectional study sampled 263 individuals divided into two groups: athletes *(n* = 121) and practitioners (*n* = 142). Their nutritional knowledge and dietary intake were measured with validated questionnaires: the Arabic Abridged Nutrition for Sport Knowledge Questionnaire and the Saudi Food Frequency Questionnaire. The majority of the sample (77.2%) had poor nutritional knowledge. Highest scoring domains for the dietary intake were protein from meat, fish, chicken (65.6%), vegetarian protein (62.4%), grains (51.0%), and the lowest were vegetables (41.1%), dairy (36.3%), and fruits (27.9%). In conclusion, athletes and practitioners in Riyadh, Saudi Arabia, have poor nutritional knowledge and dietary intake and may benefit from nutritional education and training to improve their knowledge, dietary intake, and performance.

## 1. Introduction

The number of individuals participating in athletic events and physical activities has increased in Saudi Arabia as per Vision 2030 [1]. Sports nutrition knowledge plays an important role in influencing the appropriate dietary intake, impacting athletic performance [2]. Research has shown a negative association between nutritional knowledge and supplement use [3]. Athletes and physical activity practitioners receive dietary information from social media and the internet that might not improve their performance [4]. Athletes may have some challenges in identifying the recommended macronutrient distribution [5] and may not understand the different dietary requirements that come with training, which may lead to inadequate nutritional intake [6].

Sports dietitians’ contributions have become recognized in sports settings as an integral part of high-performance teams. Their role includes promoting optimal nutrition and support and directing and teaching appropriate nutritional practices to athletes according to their training and body composition goals [7]. However, not all athletic clubs and gyms can employ qualified nutrition professionals due to budget and time limitations [8].

A systematic review of nutrition knowledge among athletes and coaches concluded that athletes lack knowledge of energy density, micronutrients, supplementation, diet sources of fat, muscle physiology, protein supplementation for vegetarians, and weight-loss management in athletes [9]. Another challenge for athletes is that their intake and nutritional requirements may not align with their perceptions of their diet [6]. Sports nutrition knowledge is necessary when athletes fail to understand how their dietary requirements change with changes in training, leading to chronically under-fueling their bodies, which may lead to injuries [6]. Athletes who participate in elite sports receive the sports nutrition education needed to optimize their performance; however, studies have found that they still have low levels of knowledge in this area [10]. The ongoing spread of misinformation regarding sports nutrition can be difficult to prevent in athletes’ environments [11,12].

Athletes and physical activity practitioners who follow specific guidelines can achieve peak performance [2]. Ergogenic aids can also enhance speed and other athletic measurements for performance; a high proportion of athletes have sub-optimal energy and carbohydrate intake [2,13]. As for the use of dietary supplements, its prevalence is high among the athletic population, especially among younger athletes and practitioners [14]. Individual and environmental factors such as knowledge, skills, peers and team culture, time, and finances can all be difficulties athletes experience when trying to consume a balanced diet and meet energy recommendations [9,15].

Evidence indicates that an inadequate understanding of fundamental nutritional principles among athletes and physical activity practitioners may result in the use of supplements endorsed through advertising campaigns [16]. Nutrition education programs in the USA, Iran, Poland, Malaysia, and Norway were found to improve the nutritional knowledge of athletes according to a systematic review [17]. In Saudi Arabia, previous studies have assessed supplement use only [18,19,20]. Only one previous study in the Middle Eastern region assessed the nutritional knowledge of Jordanian athletes and coaches through an online self-administered survey. However, that study did not assess the nutritional habits and intake, and the sample was female-dominant [21]. To the best of our knowledge, no studies have been conducted on nutritional knowledge and habits among athletes and physical activity practitioners in Saudi Arabia. This study is the first to assess nutritional knowledge, nutritional intake, and habits among athletes and physical activity practitioners in Riyadh, Saudi Arabia. And it is a step closer to determining the priorities of educational programs among this population.

## 2. Materials and Methods

### 2.1. Study Design and Population

This study is a descriptive cross-sectional study of athletes and physical activity practitioners in fitness centers and athletic clubs in Riyadh, Saudi Arabia. In this study, athletes have been defined as people who exercise to improve performance and play a particular sport in organized competitions [22]. Physical activity practitioners are individuals who participate in physical activity to increase fitness, promote health, and improve their physique [23]. The inclusion criteria for participating in this study are athletes and physical activity practitioners in Riyadh, Saudi Arabia, above 18 years old, who have agreed to participate and signed the consent form.

To have a statistically significant association between dietary intake and level of nutritional knowledge among the study’s population, we needed 264 subjects with an expected odds ratio of 2.0, with 80% power and at 0.05 level of significance [24]. Data collection started in June until December 2022, and we collected 263 subjects using convenient sampling in different athletic clubs and gyms (see Supplementary Materials Figure S1).

### 2.2. Data Collection

The questionnaire was completed during a face-to-face interview. The questionnaire consisted of five sections:

(a) Demographic information was collected first, including age, gender, social status, education level, income level, and the reason for exercise, whether they are physical activity practitioners or athletes. As well as the intensity of exercise (classification of sports by intensity levels shown in Table 1) [25], how long they have been exercising, and their source of nutritional information;
(b) Anthropometry measurements consisting of weight in kilograms and height in centimeters were documented, and Body Mass Index (BMI) was calculated;
(c) The Arabic Abridged Nutrition for Sport Knowledge Questionnaire (Arabic ANSKQ) was used to measure nutritional knowledge [21]. Response categories included agree/disagree/not sure; high/low/not sure or enough/not enough/not sure; and yes/no/not sure. Total scores were converted to percentages and classified as follows: poor (0–49%), average (50–65%), good (66–75%), and excellent (above 75%) [26];
(d) The Saudi Food Frequency Questionnaire (SFFQ) was used to assess dietary intake, use of vitamins, sports supplements, and dietary habits [27]. Frequency of consumption has been divided into 8 levels where 0 = never or less than once a month, 1 = 1–3 times a month, 2 = once a week, 3 = 2–4 times a week, 4 = 5–6 times a week, 5 = once a day, 6 = 2–3 times a day, 7 = 4–5 times a day and 8 = more than 6 times a day. And grouped into 4 frequency groups: never (0), low frequency (1,2), moderate frequency (3–5), and high frequency (6–8). The assessment of dietary intake was based on using an adapted version of the validated Australian Recommended Food Score (ARFS) [28]. A question about the use of vitamins, minerals, and nutritional supplements was used to assess the use of vitamins. One question was added regarding the use of sports supplements and ergogenic aids. For both, the name of the supplement, the dose, and the number of uses had to be specified;
(e) The physical activity levels were assessed using the Arabic version of the Global Physical Activity Questionnaire (GPAQ) [29]. The minutes per day scores were categorical (low, middle, high). The categorical score identifies categories of physical activity as low, middle, or high using the following criteria: High = vigorous-intensity activity on three days per week and accumulating to least 1500 min/week or more OR seven days per week or more of any combination of walking, moderate-intensity, or vigorous-intensity activity achieving 3000 min/week or more; Moderate = three days or more of vigorous-intensity activity totaling 60 min OR five days or more of moderate-intensity activity of 150 min or more OR five days or more of any combination of walking, moderate-intensity, or vigorous-intensity activities achieving total 600 min/week or more; Low = does not meet criteria for the moderate or high [30].

### 2.3. Statistical Analysis

Descriptive statistics described the categorical and quantitative study and outcome variables. The Kolmogorov-Smirnov and Shapiro-Wilk tests were used to test the normality of quantitative outcome variables (Nutritional knowledge scores). Data was normally distributed; the parametric tests were used in the analysis. In the Bi-variate analysis, the student’s *t*-test for independent samples and one-way analysis of variance followed by Tukey’s test of multiple comparison tests were used to compare the nutritional knowledge score in relation to the categorical study variables, which have two or more than two categories. For the categorical study variables, Pearson’s Chi-square test was used to compare the frequency distribution of food items and other variables between athletes and physical activity practitioners. Pearson’s correlation coefficient was used to quantify the relationship between two quantitative variables. In the multivariate analysis, stepwise multiple regression analysis was used to identify the independent variables related to two quantitative outcome variables: nutritional knowledge scores and sports nutritional knowledge scores. The dummy variables were created for the categorical study variables to include them in the regression analysis: physical activity intensity and levels. For physical activity intensity, the reference category was low level and was compared to medium and high levels. For physical activity level, the reference category was low level and was compared to moderate and high levels. For the groups where the subject was an athlete or a physical activity practitioner, the reference group was the physical activity practitioners’ group. A *p*-value of ≤0.05 was used to report the statistical significance of the results. Data were analyzed using Statistical Package for Social Sciences (SPSS) version 26.0 software (IBM Inc., Armonk, NY, USA).

## 3. Results

### 3.1. Socio-Demographic

A sample of 263 participants was included: 121 athletes and 142 physical activity practitioners. The subjects’ age had a mean of 26.41 ± 8.1 years. Almost two-thirds of the sample were female (60.8%) and a high proportion of subjects were single (73.4%). Approximately 66.9% of the sample held a higher education degree, while 36.9% of all participants exhibited a monthly income exceeding 8000 Saudi Riyals. Almost half of the subjects were playing high-intensity sports (47.9%). As for the duration of playing sports, more than one-third had been in sports or exercise for two years or less (34.2%), 12.6% for six to nine years, and 21.3% for more than nine years. Around a third of the athletes (33.9%) had been playing sports for three to five years, and 45.1% of the physical activity practitioners had played for two years or less. The physical activity level was low in half of the subjects (52.5%), and the remaining had high (10.6%) and moderate (36.9%) physical activity levels, whereas a high proportion of practitioners (65.5%) had low physical activity levels. The description of socio-demographic & physical exercise characteristics of study subjects is described in Table 1.

The comparison of mean values of age and BMI between athletes and practitioners shows no statistically significant difference in the mean values of age, whereas there is a significant difference in the mean values of BMI, where the mean values of BMI in athletes (22.57) are significantly lower than the mean BMI values of practitioners (24.52) (t = −3.87, *p* < 0.0001). The results indicate a significant distinction between athletes and practitioners regarding their sports intensity and level of physical activity (*p* < 0.001). More proportion in the athletes’ group were engaged in high-intensity sports and had higher physical activity levels compared to the group of practitioners, as presented in Table 2.

**Figure 1:**
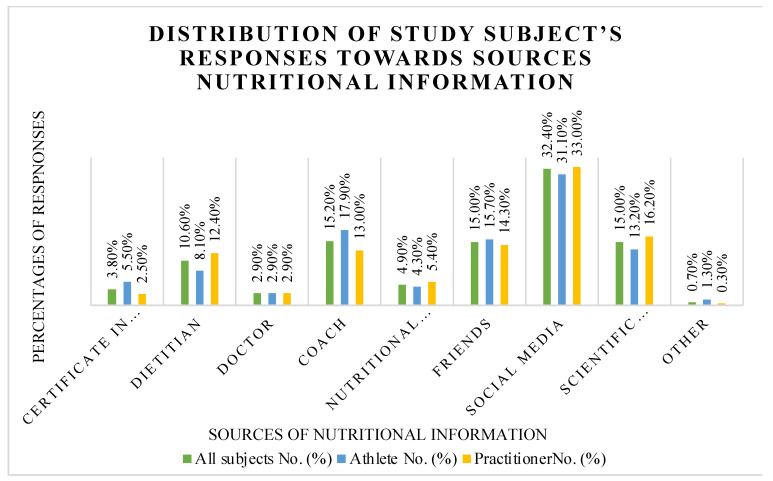
Distribution of Study Subject’s Responses Toward Sources of Nutritional Information (*n* = 546).

The sources of nutritional information were quantified as multiple responses and displayed in Figure 1. The different sources of information are: Certification in Nutrition, Dietitians, Doctors, Coaches, Nutritional books, Friends, Social Media, Scientific Sources, and other sources. Among all these sources of information, a higher proportion of athletes (31.1%) and practitioners (33.0%) selected “Social media” as their source of nutritional information, followed by coaches, scientific sources, and friends.

### 3.2. Nutritional Knowledge

The distribution of nutritional knowledge mean percentages of the study subjects among the knowledge categories is illustrated in Table 3. Total knowledge mean score showed that more than two-thirds of athletes (69.4%) and practitioners (83.8%) had poor nutritional knowledge, 25.6% of athletes and 15.5% of practitioners had fair nutritional knowledge, where only 7 subjects (6 athletes and 1 practitioner) had good total nutritional knowledge, and none had excellent knowledge. Regarding the lack of knowledge in the general knowledge section less than 10% answered correctly a question regarding the role of vitamin thiamin (B1). As for the sports nutritional knowledge, there was a substantial lack of knowledge regarding protein intake, macronutrient requirements before, during, or after training, vitamins and minerals intake, hydration, and sports supplements.

#### 3.2.1. Total Nutritional Knowledge

The comparison of mean values of total nutritional knowledge and sports nutritional knowledge in relation to the demographic and physical exercise characteristics shows statistical significance for age groups, reason for exercise, type of sports played, and physical activity levels, and are all documented in Table 4.

There is a difference in the mean total nutritional knowledge relative to the three age groups, where the mean total nutritional knowledge values are significantly higher in subjects in older age groups when compared with younger subjects (*p* = 0.028). The mean total nutritional knowledge values are higher in athletes (14.02) when compared with physical activity practitioners (13.02) (*p* = 0.040). Also, the mean total nutritional knowledge values are higher in those subjects who play medium and high-intensity sports when compared with the subjects who play low-intensity sports (*p* = 0.013), as displayed in Table 4.

#### 3.2.2. Sports Nutritional Knowledge

The mean sports nutritional knowledge values are higher in athletes (9.86) when compared with physical activity practitioners (8.53) (*p* = 0.001). The mean sports nutritional knowledge values are higher in those subjects who play medium and high-intensity sports when compared with the subjects who play low-intensity sports (*p* = 0.001). Also, the mean sports nutritional knowledge values are higher in subjects whose physical activity level is high and moderate compared to subjects with low physical activity levels (*p* = 0.019) displayed in Table 4.

#### 3.2.3. Factors Associated with Nutritional Knowledge

The Stepwise multiple regression analysis was applied to analyze the total nutritional knowledge as the dependent variable and used age, BMI, type of sports played (low intensity, medium intensity, and high intensity), and group (practitioners and athletes) as independent variables, illustrated in Table 5. The model with variables age, group, and high intensity is statistically significant (F = 6.267, *p* < 0.001). The R-square value of 0.068 indicates the 6.8% change in total nutrition knowledge scores is explained by the three variables (age, group, and high intensity). The other variables that are not statistically significant are BMI and medium-intensity sports. The coefficients of the three significant variables in the models (1.404, 0.085, and 1.139) indicate that for participants who played high-intensity sports, the total nutrition knowledge scores on average increased by 1.404 units, which compared with the subjects who played sports intensity, and for every single year increase in age, the total nutritional knowledge scores on average increased by 0.085 units, and the as for athletes, their total nutritional knowledge scores on average increases by 1.139 units when compared with the physical activity practitioners (*p* = 0.004, *p* = 0.005 and *p* = 0.018, respectively).

The Stepwise multiple regression analysis where the sports nutritional knowledge is the dependent variable and used age, BMI, type of sports played (low intensity, medium intensity, and high intensity), group (practitioners and athletes), and physical activity level (low, moderate and high) as independent variables, displayed in Table 6. The model with variables age, group, and high intensity has been shown as a statistically significant model. (F = 9.232, *p* < 0.0001). 9.7% of the change in sports nutritional knowledge scores is explained by the three variables (age, group, and high intensity). The coefficients of the significant variables in the models indicate that athletes with higher sports nutritional knowledge scores on average increase by 1.434 units compared to physical activity practitioners. In the study subjects who played high-intensity sports, their sports nutritional knowledge scores on average increased by 1.253 units compared with the subjects who played low-intensity sports, whereas for every one-year increase in age, the sports nutritional knowledge scores on average increased by 0.067 units (*p* < 0.0001, *p* = 0.002, and *p* = 0.006, respectively).

### 3.3. Dietary Intake

#### 3.3.1. Supplements

The comparison of responses between athletes and physical activity practitioners toward consuming vitamins and sports supplements showed no statistically significant difference between them *(p* = 0.730, *p* = 0.553). Where nearly half of athletes (45.5%) and practitioners (44.4%) consume some type of vitamin. The most consumed vitamins were Vitamin D, Iron, omega-3, multivitamins, and vitamin C. As for sports supplementations, only 31.4% of athletes and 25.4% of practitioners consume some type of sports supplement. Of participants who consume sports supplements, 52.8% of athletes and 51.0% of practitioners consume protein powder. As for the other types of sports supplements, 3.8% of athletes and 20.3% of practitioners consume creatine. 13.2% of athletes and 5.0% of practitioners consume pre-workout. 3.8% of athletes and 1.7% of practitioners consume energy drinks. 3.8% of athletes and none of the practitioners consume glutamine. The distribution of individuals who consume sports supplements across the intensity of sports played showed that 71.6% of those who take some type of sports supplement play high-intensity sports, 21.6% play medium-intensity sports and only 6.8% play low-intensity sports. (see Supplementary Materials Table S1).

#### 3.3.2. Dietary Habits

More than half (54.6%) of all subjects were using the oven as the usual cooking method, and 46.1% of all subjects were using olive oil as the type of fat used in cooking or baking. A similar pattern of dietary habits can be observed in both athletes and physical activity practitioners.

#### 3.3.3. Food Group Intake

The mean score for total food intake is 26.86 out of 61 points for athletes and physical activity practitioners. However, athletes (29.11) scored higher than physical activity practitioners (24.94). The highest-scoring domains were protein from meat, fish, chicken (4.59 out of 7 points), vegetarian protein (3.12 out of 5 points), grains (4.59 out of 9 points), vegetables (8.21 out of 20 points), dairy (3.27 out of 9 points), and fruits (3.06 out of 11 points), which was the lowest, and are all displayed in Table 7. Similar patterns apply to both athletes and physical activity practitioners.

#### 3.3.4. Relation between Nutritional Knowledge and Dietary Intake

Table 8 displays the correlation between sports and total nutritional knowledge scores and scoring domains of dietary intake of all study subjects, in which there is a positive correlation between protein from meat, fish, and chicken and total nutritional knowledge (r = 0.188, *p* = 0.002) and sports nutritional knowledge (r = 0.206, *p* = 0.001). There is also a positive correlation between the total intake scores and sports nutritional knowledge (r = 0.123, *p* = 0.047), whereas the scores of sports nutritional knowledge of subjects increase their total dietary intake increases.

## 4. Discussion

To our knowledge, no studies have been conducted on nutritional knowledge and habits among athletes and physical activity practitioners in Saudi Arabia. This study aimed to evaluate the nutritional knowledge and intake among physical activity practitioners and athletes in Riyadh, Saudi Arabia. Our findings contribute to the growing evidence of the essential role of nutritional knowledge in influencing food preferences and eating habits. The key findings are: (1) Most participants had poor nutritional knowledge, but athletes had better knowledge than physical activity practitioners. (2) The most frequently consumed nutrient was protein; the most frequently consumed vitamin was vitamin D; and protein powder was the most common sports supplement. (3) a positive correlation exists between total intake and sports nutritional knowledge and between protein intake and sports and total nutritional knowledge.

Total knowledge scores measured by Arabic ANSKQ have shown that around 77% of this study’s participants had poor nutritional knowledge, whether athletes or physical activity practitioners. This result is supported by the only other study that has been done in the Middle East, more specifically in Jordan, where 88.3% of their population of athletes and athletic coaches had poor nutritional knowledge [21]. Several other studies have used the longer version of the ANSKQ, the Nutritional Knowledge Questionnaire (NKQ), and concluded that athletes, irrespective of whether elite or not, had poor nutritional knowledge, where only 51% to 60.5% answered questions correctly [31,32]. In our study, the respondents appeared to lack nutritional knowledge, mainly in the sports nutrition section, in which less than 10% of them provided the correct answers in relation to protein and vitamin requirements for athletes, hydration, and supplements. There are no studies on athletes in Saudi Arabia to conduct a direct comparison.

The poor level of nutritional knowledge may be due to participants’ nutritional information source, where 31.1% of athletes and 33.0% of practitioners selected social media as their source of nutritional information. A previous study found that cross-fit trainers, high school coaches, and Australian athletes used the Internet as their primary source for nutritional information [33]. The Internet and social media could contain misinformation about nutrients and false advertisements for certain products. Interestingly, 66.9% of our sample has higher education, and only 13.2% of athletes and 16.2% of practitioners have selected scientific sources for nutritional information. 

We found a difference in the mean value of total nutritional knowledge in relation to several demographic characteristics: age groups, the reason for exercise, intensity of sports played, and physical activity levels. Regarding the reason for exercise, where athletes had better knowledge than practitioners, an explanation for this could be that athletes depend on their performance for their livelihood, and the incentive to have better knowledge and improve performance is higher in athletes than practitioners. Existing studies conducted in Jordan and Australia have reported varied findings regarding the association between demographic factors and nutritional knowledge [21,31,32].

In our study, approximately 45% of the sample consumes vitamins; most consume vitamin D, followed by iron, omega-3, multivitamins, and vitamin C. A previous study conducted among the Saudi population reported that over 60% of gym participants consume some nutrition supplement [19]. The most common types consumed do concur with our study’s findings; vitamin D, iron, calcium, multivitamins, and vitamin C were regularly consumed [20]. As for sports supplement consumption, around 28% of the sample consumed them. Previous studies on the Saudi population have suggested that younger male physical activity practitioners with the goal of bodybuilding consume more sports supplements than others. The most commonly consumed sports supplements were protein powder, amino acids, and sports drinks [19,20,34]. However, all these studies did have some methodological flaws, where no validated questionnaires were used and the sample was too small or one gender dominant, implying that results may not represent the targeted population.

The dietary intake was measured using the scoring domain adapted from a version of the validated ARFS [28], illustrating that athletes and physical activity practitioners have a mean of 26.86 out of 61 points. Several previous studies have concluded that athletes’ dietary intake does not meet the requirements and recommendations [6,7,8,35]. Jagim, et al. [6] have stated that athletes eat significantly less than the recommended for energy, carbohydrates, and protein. In addition, athletes underestimated their perceived intake of dietary fat and carbohydrates when compared to their perceived needs.

Further, the -scoring domain was meat, fish, and chicken protein. The pattern of exceeding protein requirements at the expense of carbohydrate intake has been found in several previous studies [13,36,37]. This could be due to their poor nutritional knowledge level. Athletes with insufficient energy intake might have physiological consequences such as endocrine, gastrointestinal, renal, neuro-psychiatric, musculoskeletal, and cardiovascular dysfunction called Relative Energy Deficiency in Sport [38]. This might negatively impact the health of athletes and will affect performance, including decreased endurance, increased injury risk, decreased training response, impaired judgment, decreased coordination, and decreased muscle strength [39].

Total nutritional knowledge was positively correlated with protein from the animal dietary domain. As well as we found a correlation between the total intake scores and sports nutritional knowledge. Similarly, previous studies reported an association between nutritional knowledge scores and meeting dietary requirements [7,8]. Devlin et al. [8] found, in particular, for elite athletes, higher nutrition knowledge was associated with lower protein intake, while sub-elite athletes were at the upper limit or exceeded protein recommendations. This implies that elite higher-educated athletes had protein intake closer to the recommendations [8]. A small positive correlation was found between the level of sports nutrition knowledge and carbohydrate intake for all athletes included in this study [8].

This suggests that the nutritional issues athletes face could be a result of a lack of knowledge, a misunderstanding of dietary behaviors, or the result of other negative influences and bad habits such as consuming fast foods on team trips, eating out vs. eating at home, and supplements use [6]. Altogether, our findings support the importance of providing nutrition counseling to athletes to help improve nutritional knowledge on account of the association between higher nutritional knowledge and more appropriate dietary intake, which impacts the performance and recovery of athletes. Thus, nutritional knowledge is a fundamental aspect of achieving proper nutrition. Our results gave a clear vision of where the athletic population in Riyadh, Saudi Arabia, lacks and needs in their general and sports nutritional knowledge. This contributes to getting closer to determining the priorities of educational programs among the athletic population to help with dietary intake and athletic performance.

The authors acknowledge some limitations, including its cross-sectional design, which offers only a static view of the population at a given time and cannot establish cause-and-effect relationships. Additionally, we relied on self-reported weight and height data instead of precise objective measurements. Furthermore, we did not estimate calorie and nutrient intake, and we did not exclude individuals with chronic illnesses or those taking medications from our study. Despite these limitations, this study does have strengths. Notably, it stands out as the first effort to assess the general and sports nutritional knowledge, dietary behaviors, and habits of athletes and physical activity practitioners in Saudi Arabia. Moreover, the study goes the extra mile by considering confounding factors in the stepwise regression to pinpoint factors linked to nutritional knowledge. The researchers also employed rigorous measures to ensure data accuracy, including comprehensive training for field researchers, conducting face-to-face interviews, utilizing automated data entry, and employing validated questionnaires [21,27,29]. These strengths bolster the credibility and significance of the study’s findings.

In conclusion, we found that the majority of athletes and physical activity practitioners have poor general and sports nutritional knowledge. Also, a large proportion of our sample has a moderate frequency of protein, carbohydrate, and vegetable consumption, as well as a low frequency of dairy and fruit consumption. Factors such as age, the intensity of sports played, and whether athletes or practitioners influence total and sports nutritional knowledge levels. Not meeting dietary recommendations can lead to an increased risk of injury and may alter athletic performance. This suggests that athletes and practitioners lack an appropriate understanding of basic nutritional needs that pertain to their health and performance. Those results answer the aim of understanding the nutrient intake and nutritional knowledge among physical activity practitioners and athletes in Riyadh, Saudi Arabia. This gives an insight into the current level of knowledge and intake of the athletic population, and a focus should be put on nutritional education and counseling for the athletic population, and its effect on both nutritional knowledge and dietary intake is needed. Specific questions from the ANSKQ can be used to pinpoint where the lack of knowledge lies and help improve this knowledge among the population. Coaches are an important source of nutritional knowledge to athletes and they should have more nutritional training and education. We highly recommend the availability of nutritional counseling and education programs in schools, universities, and sports settings in Saudi Arabia. This work can contribute to the establishment of sport nutrition guidelines and food and nutrition policies in recreation and sports facilities. Future studies could focus on the relationship between additional sports-specific factors, such as team versus individual sports and skills versus physique-based sports.

## Figures and Tables

**Table 1:** Classification of sports by intensity levels.

Intensity Level	
Low intensity	Cricket, golf, walking, yoga.
Medium intensity	Swimming, running, football, American football.
High intensity	Weightlifting, basketball, wrestling, tennis.

**Table 2:** Distribution of socio-demographic and physical exercise characteristics of study subjects (*n* = 263).

Characteristics		All Subjects (*n* = 263)	Athletes (*n* = 121)	Practitioner (*n* = 142)	
		**Mean and Sd**	**Mean and Sd**	**Mean and Sd**	**t-Value**
Age		26.41 (8.1)	25.61 (7.0)	27.10 (8.9)	−1.48
BMI		23.63 (4.2)	22.57 (3.5)	24.52 (4.5)	−3.87 *** ^1^
		***n* (%)**	***n* (%)**	***n* (%)**	**χ^2^-value**
Age					
	18–25	142 (54.0%)	66 (54.5%)	76 (53.5%)	
	26–35	88 (33.5%)	39 (32.2%)	49 (34.5%)	
	>35	33 (12.5%)	16 (13.2%)	17 (12.0%)	
Gender					
	Male	103 (39.2%)	55 (45.5%)	48 (33.8%)	
	Female	160 (60.8%)	66 (54.5%)	94 (66.2%)	
Social Status					
	Married	70 (26.6%)	29 (24.0%)	41 (28.9%)	
	Single	193 (73.4%)	92 (76.0)	101 (71.1%)	
Education level					
	No higher education	87 (33.1%)	48 (39.7%)	39 (27.5%)	
	Higher education	176 (66.9%)	73 (60.3%)	103 (72.5%)	
Income level					
	≤3000	73 (27.8%)	32 (26.4%)	41 (28.9%)	
	3001–8000	23 (8.7%)	11 (9.1%)	12 (8.5%)	
	>8000	97 (36.9%)	43 (35.5%)	54 (38.0%)	
	Didn’t want to answer	70 (26.6%)	35 (28.9%)	35 (24.6%)	
Type of sports played					
	Low intensity	45 (17.1%)	7 (5.8%)	38 (26.8%)	23.86 *** ^1^
	Medium intensity	92 (35.0%)	55 (45.5%)	37 (26.1%)	
	High intensity	126 (47.9%)	59 (48.8%)	67 (47.1%)	
Years of playing sports					
	≤2	90 (34.2%)	26 (21.5%)	64 (45.1%)	
	3–5	84 (31.9%)	41 (33.9%)	43 (30.3%)	
	6–9	33 (12.6%)	19 (15.7%)	14 (9.9%)	
	>9	56 (21.3%)	35 (28.9%)	21 (14.7%)	
Physical activity level					
	Low	138 (52.5%)	45 (37.2%)	93 (65.5%)	23.914 *** ^1^
	Moderate	97 (36.9%)	55 (45.5%)	42 (29.6%)	
	High	28 (10.6%)	21 (17.4%)	7 (4.9%)	

BMI: body mass index. *** ^1^ *p* ≤ 0.001.

**Table 3:** The cut-off and distribution of nutritional knowledge mean percentage of the study subjects among the knowledge categories.

Knowledge Category	Cut-Off of the Total Knowledge Mean Percentage	Total Knowledge Mean Percentage, *n* (%)	
		Athlete	Practitioner
Poor	0–49%	84 (69.4%)	119 (83.8%)
Fair	50–65%	31 (25.6%)	22 (15.5%)
Good	66–75%	6 (4.96%)	1 (0.7%)
Excellent	Above 75%	0	0

**Table 4:** Comparison of mean values of total and sports nutritional knowledge scores in relation to the demographic and physical exercise characteristics of study subjects.

Characteristics		Total Nutritional Knowledge		Sports Nutritional Knowledge	
		Mean and Sd	F-Values/t-Value	Mean and Sd	F-Values/t-Value
Age					
	18–25	12.89 (3.9)	3.607 * ^1^	8.7 (3.2)	2.923
	26–35	14.13 (3.9)		9.5 (3.2)	
	>35	14.33 (3.9)		10.0 (3.5)	
Gender					
	Male	13.13 (4.1)	1.17	8.90 (3.2)	0.982
	Female	13.71 (3.9)		9.31 (3.3)	
Social Status					
	Married	13.89 (3.8)	0.995	9.14 (3.3)	−0.016
	Single	13.34 (4.0)		9.15 (3.2)	
Education level					
	No higher education	13.28 (4.2)	−0.596	9.11 (3.3)	−0.117
	Higher education	13.59 (3.8)		9.16 (3.2)	
Income level					
	≤3000	13.33 (4.0)	2.168	9.22 (3.2)	2.252
	3001–8000	13.26 (3.3)		8.95 (2.9)	
	>8000	14.19 (3.6)		9.69 (3.2)	
	Didn’t want to answer	12.63 (4.4)		8.38 (3.3)	
Reason for exercise			2.065 * ^1^		
	Athlete	14.02 (4.3)		9.86 (3.6)	3.377 *** ^1^
	Physical activity practitioners	13.02 (3.6)		8.53 (2.8)	
Type of sports played					
	Low intensity	12.09 (3.4)	4.448 ** ^1^	7.67 (2.8)	6.822 *** ^1^
	Medium intensity	13.34 (4.0)		9.10 (3.5)	
	High intensity	14.09 (3.9)		9.70 (3.1)	
Years of playing sports					
	≤2	12.70 (3.4)	2.157	8.54 (2.8)	1.816
	3–5	14.20 (3.8)		9.67 (3.2)	
	6–9	13.61 (3.8)		9.18 (3.3)	
	>9	13.59 (4.8)		9.32 (3.8)	
Physical activity level					
	Low	13.13 (3.7)	1.292	8.64 (3.0)	4.042 ** ^1^
	Moderate	13.77 (4.1)		9.58 (3.3)	
	High	14.21 (4.6)		10.18 (3.6)	

* ^1^ *p* ≤ 0.05; ** ^1^ *p* ≤ 0.01; *** ^1^ *p* ≤ 0.001, The *t*-test was used to compare the means of two groups, and the F-test was used to compare variances of three or more groups.

**Table 5:** Stepwise multiple regression analysis between total nutritional knowledge scores and study variables.

Model	Unstandardized Coefficients				95.0% Confidence Interval for B		
	B	Std. Error	t-Value	*p*-Value	Lower Bound	Upper Bound	
1 (Constant)	12.927	0.335	38.597	<0.0001	12.267	13.587	R = 0.150 Adjusted R^2^ = 0.019 MSE = 3.920 F-test = 5.972
High intensity	1.185	0.485	2.444	0.015	0.230	2.140	
2 (Constant)	10.762	0.894	12.036	<0.0001	9.001	12.522	R = 0.218 Adjusted R^2^ = 0.040 MSE = 3.877 F-test = 6.452
High Intensity	1.399	0.487	2.875	0.004	0.441	2.357	
Age	0.078	0.030	2.607	0.010	0.019	0.137	
3 (Constant)	8.929	1.174	7.605	<0.0001	6.617	11.241	R = 0.261 Adjusted R^2^ = 0.057 MSE = 3.843 F-test= 6.267
High Intensity	1.404	0.482	2.911	0.004	0.454	2.353	
Age	0.085	0.030	2.836	0.005	0.026	0.143	
Group ^1^	1.139	0.479	2.380	0.018	0.196	2.081	

^1^ Group = Practitioner and athlete; other non-significant variables in the model are BMI and medium-intensity sports.

**Table 6:** Stepwise multiple regression analysis between sports nutritional knowledge scores and study variables.

Model	Unstandardized Coefficients				95.0% Confidence Interval for B		
	B	Std. Error	t-Value	*p*-Value	Lower Bound	Upper Bound	
1 (Constant)	7.187	0.610	11.779	<0.0001	5.986	8.389	R = 0.207 Adjusted R^2^ = 0.039 MSE = 3.193 F-test = 11.590
Group	1.348	0.396	3.404	0.001	0.568	2.128	
2 (Constant)	6.695	0.629	10.646	<0.0001	5.456	7.933	R = 0.264 Adjusted R^2^ = 0.062 MSE = 3.154 F-test = 9.698
Group	1.336	0.391	3.415	0.001	0.566	2.106	
High Intensity	1.070	0.390	2.741	0.007	0.301	1.838	
3 (Constant)	4.682	0.951	4.922	<0.0001	2.809	6.556	R = 0.311 Adjusted R^2^ = 0.086 MSE = 3.113 F-test = 9.232
Group ^1^	1.434	0.388	3.699	<0.0001	0.671	2.198	
High Intensity	1.253	0.391	3.207	0.002	0.484	2.023	
Age	0.067	0.024	2.791	0.006	0.020	0.115	

^1^ Group = Practitioner and athlete; other non-significant variables in the model are BMI and medium-intensity sports.

**Table 7:** Mean Score of food frequency questionnaire based on the Adapted Australian Recommended Food Score.

	Total (*n* = 263)		Athletes (*n* = 121)		Practitioners (*n* = 142)	
	Mean	SD	Mean	SD	Mean	SD
Total (61 points)	26.86 44.0%	12.02	29.11 47.7%	12.20	24.94 40.9%	11.57
**Scoring domains**						
Protein meat/fish/chicken (7 points)	4.59 65.6%	2.53	5.03 71.9%	2.53	4.22 60.3%	2.48
Protein vegetarian (5 points)	3.12 62.4%	1.46	3.21 64.2%	1.36	3.05 61.0%	1.53
Grains (9 points)	4.59 51.0%	2.29	4.69 52.1%	2.29	4.49 49.9%	2.30
Dairy (9 points)	3.27 36.3%	2.54	3.57 39.7%	2.60	3.02 33.6%	2.47
Fruits (11 points)	3.06 27.9%	3.26	3.43 31.2%	3.34	2.75 25%	3.17
Vegetables (20 points)	8.21 41.1%	6.26	9.17 45.9%	6.00	7.39 37.0%	6.39

**Table 8:** Correlation between sports and total nutritional knowledge scores and scoring domains of dietary intake among the study subjects.

Type of Score	Sports Nutritional Knowledge		Total Nutritional Knowledge	
	r-Value	*p*-Value	r-Value	*p*-Value
Protein from meat/fish/chicken	0.206	0.001 *** ^1^	0.188	0.002 ** ^1^
Protein vegetarian	0.000	0.994	−0.032	0.608
Grains	0.057	0.357	0.043	0.489
Dairy	0.078	0.207	0.059	0.339
Fruits	0.063	0.306	0.036	0.556
Vegetables	0.066	0.285	0.062	0.317
Total intake	0.123	0.047 * ^1^	0.099	0.111

* ^1^ *p* ≤ 0.05; ** ^1^ *p* ≤ 0.01; *** ^1^ *p* ≤ 0.001.

## Data Availability

The data presented in this study are available on request from the corresponding author. The data are not publicly available due to data protection requirements.

## Supplementary Materials

The following supporting information can be downloaded at https://www.mdpi.com/article/10.3390/nu15204353/s1, Figure S1: Data collection flow chart, Table S1: Distribution of sports supplements consumption across the intensity of sports played among study subjects.
