# Supplementary material for: Assessment of General and Sports Nutrition Knowledge, Dietary Habits, and Nutrient Intake of Physical Activity Practitioners and Athletes in Riyadh, Saudi Arabia
Source: Nutrients. 2023 Oct 12;15(20):4353. doi: 10.3390/nu15204353 (PMC10609935; doi:10.3390/nu15204353)
Supplement: Supplementary file 1 [file nutrients-15-04353-s001.zip › nutrients-2627958-supplementary.docx]

Figure S1. Data collection flowchart.

**Table S1:** Distribution of sports supplements consumption across the intensity of sports played among study subjects.

| Intensity of sports played | Consume sport supplements* | Protein powder | Creatine | Pre-workout | Energy drinks | Glutamine |
| --- | --- | --- | --- | --- | --- | --- |
| Low intensity | 5(6.8%) | 3 | 2 | 2 | 1 | 0 |
| Medium intensity | 16(21.6%) | 13 | 1 | 1 | 0 | 0 |
| High intensity | 53(71.6%) | 44 | 21 | 7 | 2 | 2 |
| Total | 74 |  |  |  |  |  |

* Multiple response
